# The Potential Impact on Farmer Health of Enhanced Export Horticultural Trade between the U.K. and Uganda

**DOI:** 10.3390/ijerph6051539

**Published:** 2009-04-28

**Authors:** Paul Cross, Rhiannon T. Edwards, Philip Nyeko, Gareth Edwards-Jones

**Affiliations:** 1 School of the Environment and Natural Resources, Deiniol Road, Bangor University, Bangor, Gwynedd LL57 2UW, U.K.; E-Mail: g.e.jones@bangor.ac.uk; 2 Centre for Economics and Policy in Health, Institute of Medical and Social Care Research, Dean Street Building, Bangor University, Bangor, Gwynedd LL57 1UT, U.K.; E-Mail: r.t.edwards@bangor.ac.uk; 3 Department of Forest Biology and Ecosystems Management, Makerere University, P.O. Box 7062, Kampala, Uganda; E-Mail: nyeko@forest.mak.ac.ug

**Keywords:** Health, malaria, migrant, vegetable production, Europe, Africa

## Abstract

The export of vegetables from African countries to European markets presents consumers with an ethical dilemma: should they support local, but relatively well-off farmers, or poorer farmers from distant countries? This paper considers the issue of farm worker health in the U.K. and Uganda, and considers the dilemma facing U.K. consumers if Uganda achieves their aim of exporting more vegetables to the U.K. Self-reported health scores of 1,200 farm workers in the U.K. and Uganda were measured with the internationally recognised SF-36 questionnaire and compared to an international population norm. The age-corrected health status of U.K. farm workers was significantly lower than the population norm, whereas Ugandans scored significantly higher (indicating good health) for physical health and lower for mental health. If Ugandan produce enters U.K. markets, then consumers may wish to consider both the potential benefits that enhanced trade could offer Ugandan farmers compared with its impacts on U.K. workers.

## 1. Introduction

Farm worker health is increasingly important as a yardstick by which consumers can evaluate the ethical merit of food producers. This growing consumer concern is implicit in the Fair Trade Movement and explicitly enshrined in the Principle of Health underpinning the International Federation of Organic Movements (IFOAM) code of good practice [1,2]. However, before consumers can make informed ethical purchasing decisions, the health status of farm workers in different systems and countries needs to be known. There are currently very few studies who report such data [3].

Supermarkets in the U.K. source their fruit and vegetable produce from a number of countries in both the developed and developing world. Developing countries may be more reliant upon this trade than the developed world because the agricultural sector tends to dominate the economy and is one of the few sectors where economic growth may be stimulated. Furthermore, successful agricultural systems in these countries play an important role in shaping the health of a population by protecting it from malnutrition and debilitating diseases. This is important as health is a factor in determining economic growth rates in developing countries [4,5]. The modernisation and growth of the agriculture sector is therefore considered as being fundamental to the improvement of the well-being of its citizens [6]. It is in this context that export horticulture has been repeatedly promulgated as a model for economic development in sub-Saharan Africa [7,8].

The economic development of a nation is important as income, health and mortality are interconnected at both the international and individual level [9]. Higher levels of social expenditure at an international level are associated with greater life expectancy [10] whilst an individual’s absolute income is a strong predictor of health status [9,11]. Thus, both Gross Domestic Product and individual income appear to be important functions of individual and population health.

The health and income relationship approximates curvilinear. Consequently changes in income levels have different effects according to income [12]. For instance, self-reported health scores can improve at a high rate per unit increase in income for individuals in the low income category, while for higher income categories increases in health status are lower per unit increase of income [13,14]. This implies that the health status of an individual from a developing country might be expected to rise by a greater amount per dollar invested than for an equivalent investment in an individual from a wealthier country.

Over recent years Kenya has developed a large export oriented horticultural sector directly or indirectly employing 500,000 workers [6,7,15], and both the urban and rural poor are thought to benefit from the greater employment stability of export horticulture. Uneducated urban women, who were previously considered unemployable, are regularly employed in pack houses in Kenya. The higher household incomes generated by export horticulture are thought to significantly reduce poverty [16].

Uganda is a neighbour of Kenya and recent studies suggest that there is considerable growth potential for export horticulture in Uganda [17,18]. Further, good agricultural performance in countries such as Uganda is thought to be a key determinant of direct pro-poor growth [19]. The lives of the poor are thought to improve in a number of ways as a consequence of economic growth. For instance, as income for the poor increases so too does their health status. Improvements in income levels are also thought to strengthen a household’s ability to cope more successfully with tropical diseases such as malaria [20,21]. If these relationships held true in Uganda, then significant economic benefits could accrue from developing an export horticulture sector.

At present the U.K. horticultural sector employs approximately 65,000 migrant workers, principally from Eastern Europe. These workers benefit from earning the U.K. minimum agricultural wage which is between 3.5 – 12 times the minimum wage of their own countries [22]. However, the economic gains appear to come at a health cost as the U.K. self-reported ill-health prevalence rate of 6,500 per 100,000 places agriculture among the highest prevalence rates of all industries [23]. Unfortunately, these figures may understate the problem for this group as many seasonally employed migrant workers only report occupation-related health symptoms upon return to their homeland [24]. This paper compares the health status of U.K. and Ugandan farm workers employed in the vegetable sector. Workers from both countries are employed to provide food for their domestic markets: in the case of the U.K., the farms supply U.K. supermarkets, whereas Ugandan workers mainly supply the inhabitants of the capital city Kampala. If Uganda were to develop an export oriented horticulture, much as Kenya did in the early 1980’s [17,18], then an increase in the availability of Ugandan produce in U.K. markets may present U.K. consumers with an ethical dilemma. The dilemma arises as consumers may wish to consider the relative social benefits arising from their support of local, U.K. horticultural businesses with those arising from their support of Ugandan businesses.

This study describes the self-reported health status of farm workers in Ugandan and U.K. vegetable horticulture and considers the impact that increased consumer purchasing of vegetables from each country may have on farm worker health.

## 2. Methods

### 2.1. Measuring Health

When measuring health the need to go beyond bio-medical indicators (such as blood pressure and cholesterol levels) has long been recognised and a raft of health questionnaires have been developed over the past 30 years to help assess the functional health status of individuals, groups and populations. Population norms have been developed for a number of these questionnaires to facilitate comparisons between groups or populations. These are benchmark scores for the general population and permit analysis of a survey sample by demographic variables such as gender and age group [25].

Four distinct health related instruments were used in this study, three of these have been widely utilised in health research: the SF-36, EuroQol EQ-5D and the Visual Analogue Scale (VAS) [25–27]. The fourth, the Short Depression Happiness Scale (SDHS), is a more recently developed instrument which has not been widely used in other studies to date. A brief description of each of these instruments is given below.

The SF-36 is an eight-scaled multidimensional health instrument that measures different attributes of an individual’s health status: physical functioning (PF), role-physical (RP), bodily pain (BP), general health (GH), vitality (VT), social functioning (SF), role-emotional (RE), and mental health (MH) [25,28]. There are two summary scales: the physical component summary scale (PCS) and the mental component summary scale (MCS): Both of which are aggregated scores for the eight scales. The SF-36 has been translated for use in over 50 countries and its results have been reported in over 4,000 publications. It has been widely accepted as a valid instrument for measuring the health related quality of life (HRQoL) for samples from the general population and those with specific health conditions [26].

U.S. national norms exist for the eight health scales and the two component scores. Scores are transformed and normalised to facilitate comparison of individual or group aggregate scores with published national norms [25,29]. Unfortunately, norms do not exist for Uganda and as a substantial proportion of the UK horticultural workforce is multi-national the 1998 U.S. national norms were used as the comparator for this instrument (http://www.SF-36.org/). This is considered an acceptable practice in multinational studies that use carefully adapted and translated HQL questionnaires [30].

The EQ-5D instrument is a generic public domain HRQoL measure in which a respondent’s health status is assessed along five dimensions (mobility, self-care, usual activities, pain/discomfort and anxiety/depression) with three separate levels of severity for each [31,32]. The U.K. EQ-5D index tariff allows scores to be compared to the U.K. population norms [27,33].

The Visual Analogue Scale (VAS) is a conceptually simple health instrument comprising a vertical line with equally spaced gradations from 0 – 100 much like a thermometer. Respondents indicate their present health status by drawing a line on the scale with the understanding that zero represents their worst possible health status and 100 their best. Population norms for the U.K. exist for this instrument [27].

The Short Depression Happiness Scale (SDHS) is a public domain instrument which allows measurements of depression and happiness across sample populations [34]. It has previously been used in the study by Cross *et al.* [3] and was included in this study as it has the potential to provide information that may be missed by the other general health instruments. No population norms exist for this instrument, although a score of 9 or below is considered potentially indicative of mild clinical depression [34].

### 2.2. Questionnaire Translation

#### 2.2.1. UK

Health questionnaires can not be assumed to be culturally invariant. The U.K. sample population was internationally diverse. Consequently, validated translations were obtained for the SF-36, EQ-5D and VAS. Questionnaires were made available to respondents in five languages English, Latvian, Lithuanian, Polish and Russian. No formally translated versions of the SDHS were available and therefore recognised, professional translators who were native speakers of the target language translated from English into Latvian, Lithuanian, Polish and Russian. No backward translation was undertaken due to resource constraints.

#### 2.2.2. Uganda

No formally translated versions of the SDHS, EQ-5D, VAS or SF-36 were available in the two principle languages used in the study districts in Uganda (Luganda and Lukonzo). Consequently, university educated native speakers of the target language translated from English to the respective target language.

### 2.3. Sample Recruitment

#### 2.3.1. UK

Due to the potential sensitivity of the research topic it was agreed with participating businesses that absolute confidentiality would be maintained about their identity. For this reason minimal descriptive data on the sample farms are presented here.

The initial U.K. project was restricted to large farms supplying brassicas, peas, beans, onions, leeks, lettuce and endives to U.K. supermarkets. Farm businesses in the U.K. were identified and recruited through pre-existing contacts with the researchers as well as through telephone listings and web sites. Contact with the businesses was established by phone followed up with meetings with farmers and/or managers if they were willing.

For the purposes of sample identification fieldworkers were understood to be those members of staff, employed either on a seasonal or permanent basis, who worked primarily in the field. Typical work tasks for this group included planting, harvesting, weeding or crop spraying as well as those who supervised the workers or drove tractors in the field. Packhouse workers were defined as anyone working extensively in the packhouse performing tasks involving grading, packing, stacking, tray-lining, washing or tractor work within the packhouse or warehouse areas.

The dissemination of questionnaires was undertaken at two distinct times (July and August in 2006, April and May in 2007) with the cooperation and coordination of the human resources department of the larger farms, or through the farm owner on smaller farms. All score comparisons between the U.K. and Uganda refer to the U.K. data collected in 2006. The smaller 2007 data set was used uniquely for comparisons between a sample of the 2007 cohort at the start of the season (and their employment) and mid-season farm worker scores for 2006.

#### 2.3.2. Uganda

The Ugandan survey frame was restricted to farmers and farm workers cultivating one or more of the vegetable groups identified in the U.K. sample. This was done in an attempt to limit the impact of differing cultivation regimes on farm worker health through the use of differing crop treatment chemicals.

Ugandan farms were identified by research collaborators at Makerere University. Extension officers were then allocated to the research team to act as facilitators and guides in the survey areas. Prior permission to interview farm workers was obtained from the farm owner by the extension officers.

Three of the survey districts selected in Uganda were Mukono, Wakiso and Luwero. They were chosen because of their proximity to Kampala and were all within a two hour drive of Entebbe international airport. The fourth sampling location was Kasese situated in the west of the country near to the border of the Democratic Republic of Congo. The area contains a long established irrigation project which produces a wide variety of vegetables principally for the Kampalan market. The sample farms were identified with the participation of relevant extension officers in liaison with the Department of Forest Biology and Ecosystems Management, Makerere University. Two trained research assistants undertook face to face interviews with farm workers of adult age of both sexes in the field.

### 2.4. Data Analysis

Differences between groups were analysed using non-parametric Mann-Whitney *U* test, Kruskal-Wallis and student t-tests. Where appropriate, associations between mean scale scores were explored using Spearman’s rank correlations. Differences between groups and population norms were investigated using student t-tests. The Ugandan health scores were compared with the U.K. farm worker health scores published in Cross *et al.* [3]. Where appropriate both Ugandan and U.K. SF-36 scores were compared with the U.S. population norms.

Multiple regression analysis was used to investigate the relationship between self-reported health status and twelve potentially relevant variables (house type, malaria within the past three months, distance travelled to work, number of children per respondent, whether the respondent smoked or had smoked in the past, level of education, annual income, bicycle ownership, radio ownership, mobile phone ownership, job status and number of tasks performed each day).

Candidate variables were entered into a backward stepwise elimination model to explore variation within PCS and MCS scores. Multicollinearity can be problematic when including a large number of variables in the analysis as parameter variance and the r^2^ value can tend to increase leading to an increased probability of committing a type II error [35]. Consequently, multicollinearity was tested by setting the tolerance value at less than 0.2 and the Variance Inflation Factor (VIF) considerably less than 5.

## 3. Results

### 3.1. Sample Description

#### 3.1.1. UK

Eight U.K. farm businesses were recruited to the study. Five were conventional farms, one was organic and the remaining two comprised both conventional and organic aspects. The farms were geographically separate from each other and staff did not move between them. Four of the larger farms employed between 100 and 1,500 workers with a fifth employing only 15. The three smaller farms were family run.

Of the approximately 1,250 questionnaires distributed to workers, only 698 were returned, giving a response rate of approximately 56%. Subsequent to a triage of the questionnaires, whereby incomplete or incorrectly completed copies were rejected, the final number of completed questionnaires by field and packhouse workers was 605. The sample comprised 395 males and 210 females. The majority of field and packhouse workers (93%) employed on survey farms were of non-British nationality (British (42), Bulgarian (68), Estonian (1), Latvian (24), Lithuanian (156), Moldovan (28), Polish (123), Romanian (2), Russian (28), Slovakian, (2), South African (5), Ukrainian (126)).

The marital status category of the questionnaire allowed four possible responses; single (79%), married/partnered (20%), divorced (0.6%) and widowed (0.4%). Fourteen percent of the respondents said they had children and of these, 63% had at least one child less than five years of age. Three responses were possible for the ‘do you smoke’ question; smoker (28%), ex-smokers (10%) and never smoked (62%).

#### 3.1.2. Uganda

A total of 571 questionnaires were administered to individual farmers and farm workers in Uganda through personal interviews. The sample population comprised 282 males and 289 females, sampled from 62 farms supplying vegetable produce to Kampala. Most of the workers were field workers, with the exception of those working in the Kasese district where a number of the workers were employed in a packhouse. Whilst one of the farms in this district was organic, the workers were transitory and worked on conventional farms as well.

The marital status category of the sample was: single (16%), married/partnered (66%), divorced (8%) and widowed (10%). Eighty-five percent of the respondents said they had children and of these, 50% had at least one child less than five years of age. The average number of children per respondent was 3.5. Three responses were possible for the ‘do you smoke’ question; smoker (7%), ex-smokers (4%) and never smoked (89%). Malaria was the only serious illness explicitly mentioned by respondents, 37% of whom claimed to have experienced an episode in the three months preceding the survey.

### 3.2. Health Scale Correlations

All scales of the SF-36, EQ-5D, VAS and the SDHS were highly significantly correlated in both the U.K. and Uganda (*p* < 0.0001) although inter-scale correlations for the Ugandan sample were stronger than those found in the U.K. study which gives some degree of confidence concerning the translations. As a consequence of the strength of the correlations between the EQ-5D, VAS and SDHS only the SF-36 scores are reported in the results.

### 3.3. Comparison of UK Scores with US Norms

More than 95% of the U.K. sample population were aged between 18 and 34 and consequently only scores for this age group are reported here. The 18–34 population scores were significantly higher than the US population norm for vitality (VT) only and significantly lower for role-physical (RP), bodily pain (BP), general health (GH) social-functioning (SF), mental health (MH) and the physical component summary score (PCS) (Table 1).

### 3.4. Comparison of Ugandan Scores with U.S. Norms

The overall population scores were significantly higher than the U.S. population norm for physical functioning (PF) and the physical component summary score (PCS) and significantly lower for role-physical (RP), role-emotional (RE), mental health (MH) and the mental component summary score (MCS).

When the scores for those workers who had suffered malaria in the three months preceding the survey were removed from the sample the overall population scores were higher than the U.S. population norm for physical functioning (PF), bodily pain (BP), general health (GH), vitality (VT), social functioning (SF) and the physical component summary score (PCS). They remained significantly lower for role-physical (RP), role-emotional (RE), and the mental component summary score (MCS) (Table 2).

**Figure 1.**
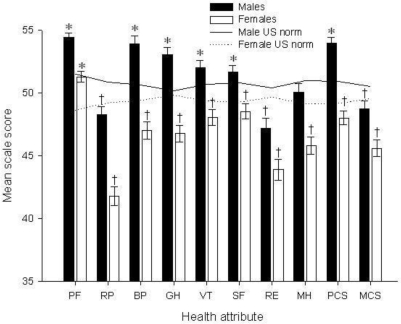
Ugandan SF-36 scores by gender. Physical Functioning (PF), Role-Physical (RP), Bodily Pain (BP), General Health (GH), Vitality (VT), Social-Functioning (SF), Role-Emotional (RE), Mental Health (MH), Physical Component Summary (PCS), Mental Component Summary (MCS). * Ugandan farm worker scale scores were significantly higher than the population norm. † Ugandan farm worker scale scores were significantly lower than the U.S. norm.

Ugandan males scored significantly higher than females for all SF-36 scales (Kruskall Wallis, df = 1, *p ≤* 0.001) although the role-emotional scale (RE) significance value was less (df = 1, *p* = 0.002) (Figure 1). Males scored significantly higher than the U.S. population norms for physical functioning, bodily pain (BP), general health (GH), vitality (VT) and the physical component summary scale (PCS) and significantly lower for role-physical (RP), role-emotional (RE), mental health (MH) and the mental component summary scale (MCS) (Table 2). Ugandan female farm worker scale scores were significantly higher than the U.S. norms for physical functioning (PF) and significantly lower for all other SF-36 scales.

When the Ugandan scale scores were controlled for by age the 18 – 34 age group was significantly higher than the corresponding U.S. norms for vitality (VT) and significantly lower for role-physical (RP), role-emotional (RE), and the physical component summary score (PCS). When the scores for those workers who had suffered malaria in the three months preceding the survey were removed, scores were significantly higher than the U.S. population norms for bodily pain (BP), general health (GH), vitality (VT) and remained significantly lower for role-physical (RP) and role-emotional (RE) (Table 2).

### 3.5. Comparisons of Scores between the U.K. and Uganda for the 18–34 Age Group

As 96.5% of the U.K. sample was aged 18 – 34 the following comparisons between scores for the U.K. and Uganda refer solely to this age group. Ugandan farm worker 18–34 scores were significantly higher than the corresponding U.K. scores for physical functioning (PF), bodily pain (BP), general health (GH), social functioning (SF) and mental health (MH) and significantly lower for role-emotional (RE).

When the scores for those workers who had suffered malaria in the three months preceding the survey were removed Ugandan farm workers scored significantly higher than U.K. farm workers for physical functioning (PF), bodily pain (BP), general health (GH), social functioning (SF), mental health (MH), the physical component summary scale (PCS) and the mental component summary scale (MCS) scores of Ugandan farmers were not lower than the U.K. workers on any scale (Table 3).

### 3.6. Contribution of Socio-Demographic Variables to Health Scores

In an attempt to better understand the relative contribution of different socio-demographic and occupational factors to U.K. and Ugandan health, the PCS and MCS scores were utilised as dependent variables in a multiple linear regression model. Independent variables entered into the first model for the U.K. were farm, farm size, farming method, number of tasks per day, wages, age, gender, nationality, marital status and children. A significant model emerged for the PCS (*F_4,421_* = 7.64, p ≤ 0.001, adjusted r^2^ = 0.059) with the significant variables being tasks (β = 0.153, p = 0.001), marital (β = −0.17, p = 0.003), children (β = −0.127, p=0.027) and farm (β= −0.179, p ≤ 0.001). A significant model also emerged for MCS (*F_4,421_* = 9.799, p ≤ 0.001 adjusted r^2^ = 0.076). Significant variables were farming method (whether the farm worker worked on an organic or conventional farm β = 0.134, p = 0.011) children (β = −0.133, p = 0.005) farm (β = −0.186, p ≤ 0.001) and farm size (farm size was measured by the number of seasonal employees β = −0.228, p ≤ 0.001).

Independent variables entered into the first model for Uganda were house type, malaria within the past three months, distance traveled to work, number of children per respondent, whether the respondent smoked or had smoked in the past, level of education, annual income, bicycle ownership, radio ownership, mobile phone ownership, job status and number of tasks performed each day at work. A significant model emerged for the PCS (*F*_5,504_ = 18.86, *p* ≤ 0.001, adjusted r^2^ = 0.149), with the significant variables being education (β = −0.132, *p* = 0.002), annual income (β = 0.23, *p* ≤ 0.001), malaria (β = −0.119, *p* = 0.004), number of tasks (β = 0.179, *p* ≤ 0.001) and house type (β = 0.091, *p* ≤ 0.033). A significant model also emerged for the MCS (*F*_5,504_ = 10.633, *p* ≤ 0.001, adjusted r^2^ = 0.086). Significant variables were smoking (β = 0.088, *p* ≤ 0.039), annual income (β = 0.084, *p* ≤ 0.05), malaria (β = −0.204, *p* ≤ 0.001), travel (β = −0.14, *p* = 0.001) and house type (β = 0.09, *p* = 0.037).

**Figure 2.**
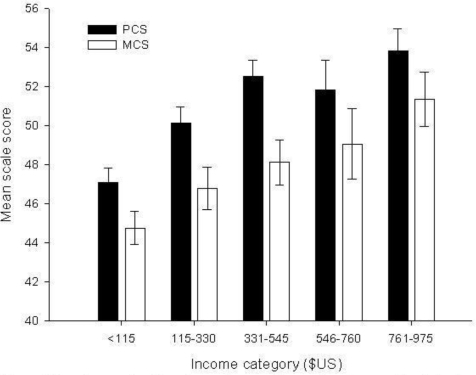
Ugandan mean health scale score by annual income class for mean Physical and Mental Component Summary scores (PCS and MCS).

The mean self-reported annual income per capita was $US 398, with males earning more than twice that of females (males $US 553, females $US 248). Ninety one percent of the sample population earned less than $US 1000 per annum. The incomes of these workers were aggregated into five income category groups to explore possible relationships between health scores and income categories. The mean PCS and MCS scores differed significantly between annual income classes (PCS df 3 *p* ≤ 0.001; MCS df 3 *p* ≤ 0.001) (Figure 2). As income increased so did the mean score for the health scales. Annual income in Uganda differed significantly with respect to the level of educational attainment (n = 437 *p* = 0.02). Mean annual income for those who attended primary school was $US 347 compared to $US 455 for those who attended secondary school.

The type of house occupied by workers appeared to be a function of their annual income. There were significant differences in house type dependent upon income levels (Mann Whitney n = 522, *p* ≤ 0.001). Those who described their house as mud and wattle (n = 204, mean annual income $US 275) had an annual income almost half that of those who owned a brick house (n = 319, mean annual income $US 475, Kruskal Wallis df = 1, *p* ≤ 0.001).

Bicycle ownership had a significant impact on both mental and physical scales (Mann-Whitney U PCS n = 565, *p* = 0.003; MCS n = 565, *p* = 0.015). The mean cost of a bicycle was $US 44.25. The positive effect on health scores was more marked if the respondent owned a radio (Mann-Whitney U, PCS n = 561, *p* ≤ 0.001; MCS n = 561, *p* ≤ 0.001). The mean cost of a radio was $US 22.13.

### 3.7. Longitudinal Assessment of Migrant Workers in the U.K. during 2007

All SF-36 scale and component summary scores (with the exception of physical functioning (PF)) were significantly higher for U.K. farm workers at induction for the 2007 cohort than those recorded for workers mid-season in 2006 (Table 4). The sample was drawn from the four largest farms used in the survey of 2006. There were no significant differences in the gender or age composition for each cohort.

## 4. Discussion

Ugandan male farm workers scored significantly higher than both females and the U.S. population norm for all SF-36 scales except for physical functioning. This is indicative of better health. This reflects similar findings in a study in Tanzania where males scored higher than females for all SF-36 scales [36].

Ugandan and U.K. farm workers showed no significant differences between their SF-36 physical and mental component summary scores although there were differences for particular sub-scales. The absence of difference between the two workforces may be a reflection of the poor mid-season health status of U.K. farm workers rather than an indication of Ugandan good health as the scores for U.K. workers appears to decline during the season whereas Ugandan farm workers are constant. However, when those Ugandan respondents who reported malaria were removed the scores were higher for a number of SF-36 scales and not lower on any. Similarly Ugandan workers aged 18 – 34 had similar scores to the U.S. population once malaria sufferers had been removed. The Tanzanian urban dwellers in the study by Wagner *et al.* [37] also had similar scores to the U.S. population when age differences were accounted for [37].

It is important to note that the method of data collection may have influenced the results. Ugandan respondents may have reported better health as they were interviewed face-to face whereas the U.K. workers completed the questionnaire alone and in their own time. Only face to face interviews were viable in Uganda due to the high levels of illiteracy. Studies in the U.S. and Australia have shown that respondents tend to report better emotional and physical health in interviews [38,39]. However, the magnitude of the differences between the health scores of Ugandan farm workers and the age controlled population norm and U.K. scores appears to be of a magnitude that cannot easily be explained solely by the interview technique. A more pertinent question may be to investigate why farm workers health scores in the U.K. appear to decline so strongly during the season.

### 4.1. Wider Implications

There is now an established relationship between income level and health status [12]. As income for a population increases so too does their health status, although at an ever diminishing marginal rate. The relationship implies that the greatest improvement in population health for a unit increase in income would be expected to accrue to the lowest income workers. The Ugandan farm worker data appears to suggest that the relationship between income and health is still positive and linear whereby a unit increase in income corresponds to an equivalent increase in good health status (Figure 2). It is interesting to note that the U.K. health scores were largely independent of income. This suggests that attempts to improve worker health through the lever of increased income are likely to be limited. Previous analysis of health drivers in the U.K. horticultural workforce identified the monotonous nature of the work and possibly workers’ material living conditions and homesickness as being primarily responsible for poor health [3]. By contrast Ugandan workers’ health appears to be much more sensitive to income.

Whilst self-reported health is commonly used to measure a sample population’s health status, it has also been frequently employed as an indicator of mortality rates [40,41]. In these studies, respondents were asked to rate their health as very good, good, fair, poor or very poor (the equivalent in the SF-36 is excellent, very good, good, fair and poor). Follow up studies suggested that those answering ‘poor’ or ‘very poor’ had a subsequent increased risk of mortality compared to those answering ‘good’ or ‘very good’. For example, in the Whitehall study Singh-Manoux *et al.* [41] found that 3.7% of middle-aged men and 7.1% of middle-aged women described their health as poor or very poor. Their subsequent mortality rate was 3.8 times higher over a ten year period than those who described their health as good or very good. In a further study of middle-aged British males [42], those reporting poor health had a mortality rate of 45 deaths per thousand compared with 5.5 deaths per thousand for those reporting excellent health. This equated to an eight fold increase in mortality per 1,000 per year.

In the present study, the proportion of U.K. and Ugandan farm workers aged 18 – 34 describing their health as poor or fair (the lowest two categories) was 14% and 18% respectively. If the relationship, as described by Singh-Manoux [41] and Wannamethee and Shaper [42], between an individual’s self-reported poor health and subsequent mortality rate holds, then these individuals may have an increased risk of mortality. The proportion of individuals in this category is far greater than that reported by Singh-Manoux [39] and Wannamethee and Shaper [42].

### 4.2. Agriculture and Tropical diseases: The Case for Malaria

After controlling for the effects of malaria the Ugandan farm workers’ scores were significantly higher than those of the U.K. workforce and not significantly different to the U.S. population norm. Income and malaria were important explanatory variables in the multiple regression analysis. The causality appears to be bidirectional with income levels correlated with malarial infection rates and malarial incidence impacting income levels [43]. The importance of this relationship is borne out by a number of studies that suggest that there is an important financial cost incurred following malaria illness [44] which can impact upon a household’s ability to maintain living standards [21,45]. Thus, the potential of economic improvement at a local scale to positively influence the health status for farmers and farm workers in Uganda appears to be very large. This is in contrast to the expected health problems that accrue in the short-term to East European workers in the U.K.

### 4.3. Ethical Considerations

There are a number of considerations that may need to be evaluated with regard to the ethical appropriateness of continuing to grow vegetables in the U.K., which extend beyond the ‘food miles’ debate [46]. For instance, health costs incurred as a consequence of working in the U.K. may ultimately have to be borne by the workers’ home country. In addition migrant workers may gain a financial benefit from working in the U.K. but the extent to which this compensates for a decline in health remains unexplored.

Consumers whose preferential purchasing of British produce is based upon notions of ethics may wish to consider the relevance of human health as a factor in their purchase decisions for two reasons. Firstly, when British produce is bought from a U.K. supermarket the perceived health status of a worker declines, at least in the short-term. Secondly, there are potential alternative production centres outside the U.K. meriting further consideration such as Uganda, where health may improve as a consequence of consumers purchasing. If such comparative poor health is due to the U.K. working conditions then the ethical permissibility of such a system needs to be tackled on a number of fronts. For instance, ‘local food’ proponents may wish to consider how increased local food production at a local scale may impact the health of workers in the local food chain. Furthermore, managers on horticultural farms will need to respond to the fact that worker health declines during the season. Attempts to redress the problem may require the adoption of new working practices that can counteract these effects, such as offering a diversity of tasks in the workplace, as well as improving the employees’ social environment.

It is against this backdrop that the topic of farm worker health is becoming increasingly prominent in food production debates particularly as movements such as Fairtrade and IFOAM re-evaluate concepts of agricultural social justice. The factoring in of the costs of poor health to the farmer and society as a whole needs to be considered by policy makers for both the short and long-term.

Long-term health costs may be difficult to detect, particularly for those workers who return to their home country and receive medical care at a later date. Costs may be incurred by the donor country and the extent to which this would be morally acceptable remains unexplored. At a European level the cost of palliative care in one country may be compensated for by the health benefits derived from increased vegetable consumption in another country. For instance, if a Polish worker experiences a decrease in health status of one unit for every ten thousand lettuces that he or she harvests, consumers’ health status may need to increase by an equivalent amount through the consumption of lettuce in order to balance the population health status.

Further research should assess the costs and benefits that accrue to migrant workers working in U.K. horticulture, and how these impact their health following their return to their homeland. In addition it would be useful to work with farm managers in order to alter work patterns such that worker health improves. Meanwhile in developing countries considerable work is needed to fully understand the relationship between income, participation in the export market and farmer health.

## Figures and Tables

**Table 1.** U.K. t-test scores for the SF-36 for the 18 – 34 disaggregated by age category and gender compared to the published population norm. Physical Functioning (PF), Role-Physical (RP), Bodily Pain (BP), General Health (GH), Vitality (VT), Social-Functioning (SF), Role-Emotional (RE), Mental Health (MH), Physical Component Summary (PCS), Mental Component Summary (MCS).

	**PF**	**RP**	**BP**	**GH**	**VT**	**SF**	**RE**	**MH**	**PCS**	**MCS**
General (df 901)	0.1649	0.001^a^	< 0.0001^a^	0.002^a^	0.0003^b^	< 0.0001^a^	0.8893	0.0001^a^	0.0002^a^	0.0979
Males (df 413)	0.0442	0.005^a^	0.0003^a^	0.0197^a^	0.1084	< 0.0001^a^	0.4111	< 0.0001^a^	0.0048^a^	0.0123^a^
Females (df 354)	0.4467	0.1864	0.0016^a^	0.1591	0.0044^b^	0.0418^a^	0.3837	0.714	0.7234	0.5444

^a^ Scores were significantly lower than U.S. norms.

^b^ Scores were significantly higher than U.S. norms.

**Table 2.** Ugandan farm worker scores disaggregated by gender, age and malaria for the SF-36 scales Physical Functioning (PF), Role-Physical (RP), Bodily Pain (BP), General Health (GH), Vitality (VT), Social-Functioning (SF), Role-Emotional (RE), Mental Health (MH), Physical Component Summary (PCS), Mental Component Summary (MCS).

**Characterisation details of Ugandan samples compared with U.S. population norms**												
Males				Females			Total			Malaria control		
Scale	Mean	df	p	Mean	df	p	Mean	df	p	Mean	df	p
PF	54.44	1064	< 0.001^a^	51.28	1486	< 0.001^a^	52.84	2552	< 0.001^a^	53.45	2372	< 0.001^a^
RP	48.25	1064	<0.001^b^	41.85	1486	< 0.001^b^	45.01	2552	< 0.001^b^	46.81	2372	< 0.001^b^
BP	53.92	1063	< 0.001^a^	47.07	1486	< 0.001^b^	50.44	2551	0.312	52.34	2372	< 0.001^a^
GH	53.06	1063	< 0.001^a^	46.82	1484	< 0.001^b^	49.90	2549	0.662	51.57	2370	0.002^a^
VT	51.98	1062	0.025^a^	48.12	1485	< 0.001^b^	50.02	2549	0.962	51.59	2371	0.002^a^
SF	51.65	1064	0.124	48.54	1485	0.015^b^	50.07	2551	0.866	51.38	2371	0.006
RE	47.20	1064	<0.001^b^	43.94	1486	< 0.001^b^	45.54	2552	< 0.001^b^	47.06	2372	< 0.001^b^
MH	50.02	1062	0.009^b^	45.86	1485	< 0.001^b^	47.91	2549	< 0.001^b^	49.50	2371	0.029^b^
PCS	53.95	1060	< 0.001^a^	48.03	1484	0.002^b^	50.94	2546	0.022^a^	52.23	2370	< 0.001^a^
MCS	48.72	1060	<0.001^b^	45.64	1484	< 0.001^b^	47.15	2546	< 0.001^b^	48.63	2370	< 0.001^b^

**Characterisation detailsof Ugandan farm workers aged 18–34 with U.S.population norm**						
	Total 18 – 34			Malaria control (18–34)		
Scale	Mean	df	p	Mean	df	p
PF	54.19	629	0.745	54.60	552	0.232
RP	46.28	629	< 0.001^b^	48.06	552	<0.001^b^
BP	52.05	629	1.000	53.65	552	0.018^a^
GH	51.70	628	0.566	53.06	552	0.008^a^
VT	51.08	628	0.002^a^	52.80	552	<0.001^a^
SF	50.57	629	0.830	51.40	552	0.146
RE	45.69	629	< 0.001^b^	47.85	552	<0.001^b^
MH	48.76	627	0.590	50.24	552	0.103
PCS	52.74	627	0.007^b^	53.64	552	0.755
MCS	47.26	627	0.166	49.00	552	0.160

^a^ Ugandan farm worker scale scores were significantly higher than U.S. population norm.

^b^ Ugandan farm worker scale scores were significantly lower than the U.S. population norm. Malaria control = scores were removed from the analysis for workers who self-diagnosed as suffering from malaria in the three months preceding the survey.

**Table 3.** Comparison of the U.K. and Ugandan scores for the SF-36 scales Physical Functioning (PF), Role-Physical (RP), Bodily Pain (BP), General Health (GH), Vitality (VT), Social-Functioning (SF), Role-Emotional (RE), Mental Health (MH), Physical Component Summary (PCS), Mental Component Summary (MCS).

		**PF**	**RP**	**BP**	**GH**	**VT**	**SF**	**RE**	**MH**	**PCS**	**MCS**
**18–34 age group**	U.K.	50.26	47.27	48.37	49.37	51.48	45.93	47.95	46.43	51.97	46.79
	Uganda	54.19	46.28	52.05	51.70	51.08	50.57	45.69	48.76	52.74	47.26
	*df*	826	826	797	779	789	794	804	78a8	748	748
	*p*	< 0.001^a^	0.091	< 0.001^a^	< 0.001^a^	0.397	< 0.001^a^	< 0.001^b^	< 0.001^a^	0.081	0.470
**Malaria control 18–34 age group**	U.K.	50.26	47.27	48.37	49.37	51.48	45.93	47.95	46.43	51.97	46.79
	Uganda	54.6	48.06	53.65	53.06	52.8	51.4	47.85	50.24	53.64	49
	*df*	749	749	720	703	713	717	727	713	673	673
	*p*	< 0.001^a^	0.442°	< 0.001^a^	< 0.001^a^	0.088°	< 0.001^a^	0.867°	< 0.001^a^	< 0.001^a^	0.003^a^

(^a^) Ugandan farm worker scale scores were significantly higher than U.K.

(^b^) Ugandan farm worker scale scores were significantly lower than the U.K. scores. Malaria control = scores were removed from the analysis for workers who self-diagnosed as suffering from malaria in the three months preceding the survey.

**Table 4.** Comparison of U.K. farm worker health scores at the beginning of service in 2007 and mid-season 2006. Means were compared using the Mann Whitney *U* test. Farm worker mid-season 2006 scores were significantly lower for all health scales than the induction scores for 2007.

	**Induction 2007**			**2006**			
	Mean	n	S.D.	Mean	n	S. D.	*p*
PF	54.94	193	4.42	54.1	395	7.78	0.3612
RP	53.2	193	6.28	50.26	395	8.55	< 0.0001
BP	54.79	193	8.8	48.46	395	10.54	< 0.0001
GH	51.57	193	8.08	49.16	395	8.99	0.0031
VT	57.05	193	7.84	51.38	395	9.67	< 0.0001
SF	51.74	193	7.15	46.69	395	10.41	< 0.0001
RE	52.88	193	6.38	49.97	395	9.27	0.0003
MH	51.39	193	8.88	46.1	395	10.6	< 0.0001
PCS	54.46	193	4.82	52.07	395	6.78	< 0.0001
MCS	52.03	193	7.7	46.71	395	9.98	< 0.0001
